# The relationship between repeated measurements of HbA_1c_ and risk of coronary events among the common haptoglobin phenotype groups: the Action for Health in Diabetes (Look AHEAD) study

**DOI:** 10.1186/s12933-024-02448-z

**Published:** 2024-10-09

**Authors:** A. S. Carew, R. A. Warren, M. P. Bancks, M. A. Espeland, J. L. Bahnson, C. L. Lewis, A. P. Levy, J. L. Sapp, R. Urquhart, J. L. Wang, E. B. Rimm, L. E. Cahill

**Affiliations:** 1 QEII Health Sciences Centre, Nova Scotia Health, Halifax, Canada; 2 Department of Community Health and Epidemiology, Dalhousie University, Halifax, Canada; 3 Department of Medicine, Dalhousie University, Halifax, NS Canada; 4 Department of Epidemiology and Prevention, Wake Forest University School of Medicine, Winston-Salem, USA; 5 Department of Biostatistics and Data Science, Wake Forest University School of Medicine, Winston-Salem, USA; 6 Department of Internal Medicine-Gerontology and Geriatric Medicine, Wake Forest University School of Medicine, Winston-Salem, USA; 7 Department of Epidemiology, University of Alabama Birmingham, Birmingham, USA; 8 Rappaport Faculty of Medicine, Technion Israel Institute of Technology, Haifa, Israel; 9 Department of Epidemiology, Harvard T. H. Chan School of Public Health, Boston, USA; 10 Department of Nutrition, Harvard T. H. Chan School of Public Health, Boston, USA

**Keywords:** Coronary artery disease, Epidemiology, Genetic association, Glycated hemoglobin, Haptoglobin phenotype, Type 2 diabetes mellitus

## Abstract

**Background:**

In the ACCORD study, participants with the haptoglobin (Hp) 2–2 phenotype and glycated hemoglobin (HbA_1c_) ≥ 8.0% had a higher risk of coronary artery disease (CAD) compared to those with HbA_1c_ 7.0–7.9%. However, this association was not observed in participants without the Hp2-2 phenotype. The optimal glycemic target for CAD prevention for the Hp phenotypes remains uncertain and may vary based on demographic and clinical factors.

**Objective:**

To investigate how reaching clinically relevant HbA_1c_ targets relates to the risk of CAD in different Hp phenotype groups among a diverse cohort of individuals with T2DM (the Look AHEAD study, HbA_1c_ ≤ 11% at baseline).

**Methods:**

Cox regression models with time-varying covariables were used to quantify the association between time-varying achieved HbA_1c_ (< 6.5%, 6.5–6.9%, and ≥ 8.0% compared to 7.0-7.9%), updated at years 1–4, 6, 8, and 10, and incident CAD in the Hp2-2 (*n* = 1,587) and non-Hp2-2 (*n* = 2,944) phenotypes separately. Further pre-specified subgroup analyses by age, sex, history of cardiovascular disease (CVD), race, and diabetes duration were performed in each Hp phenotype group separately.

**Results:**

Compared with HbA_1c_ 7.0-7.9%, having HbA_1c_ < 6.5% was associated with a 29% lower CAD risk among participants with the non-Hp2-2 phenotype (adjusted HR 0.71, 95% CI 0.55–0.90). In subgroup analyses, this association was present in participants with the non-Hp2-2 phenotype who were male (0.60, 0.44–0.83), who did not have a history of CVD (0.65, 0.47–0.90), who were aged ≥ 65 years (0.64, 0.44–0.94), who were White (0.68, 0.51–0.91), or who had diabetes duration > 10 years (0.58, 0.35–0.95). HbA_1c_ ≥ 8.0% was associated with CAD risk only among participants with the Hp2-2 phenotype who had a history of CVD (1.79, 1.00-3.20). No associations were found between the other HbA_1c_ targets and CAD risk when participants with the Hp2-2 phenotype were grouped together or divided into subgroups.

**Conclusion:**

The differences in our results compared to our previous findings may be due to variations in the study populations and factors associated with weight loss, making it difficult to draw definitive conclusions. Our current findings should be considered in the context of hypothesis generation, and ideally, will encourage additional research in this field.

**Supplementary Information:**

The online version contains supplementary material available at 10.1186/s12933-024-02448-z.

## Introduction

Optimal glycemic control is an integral component of type 2 diabetes mellitus (T2DM) management for the prevention of the common and costly complication of coronary artery disease (CAD). However, a frequent adverse effect of intensive glycemic control is hypoglycemia [1–3], which is associated with a reduction in health-related quality of life, increased health care costs, and an increased risk of death [4–7]. Additionally, intensive glycemic control can result in more adverse drug reactions, increased treatment burden, and higher healthcare costs for patients due to the need for multiple medications [8]. Several diabetes organizations have released recommendations to healthcare providers emphasizing the importance of personalizing glycemic goals and treatment plans for patients with T2DM, focusing on person-centered care and shared decision-making [9–11]. Current clinical practice guidelines recommend a glycemic target of < 7.0% for most adults with T2DM [9, 12, 13]. However, less stringent targets are advised for individuals with advanced age, frailty, limited life expectancy, long-standing diabetes, advanced atherosclerosis, comorbidities, and a known history of severe hypoglycemia [9, 10]. Our previous research has identified haptoglobin (Hp) phenotype as an additional characteristic that could be used to personalize glycemic targets for people with T2DM to prevent CAD [14–16].

Studies have shown that a common variation in the gene that codes for the Hp protein identifies individuals who may be at increased risk of CAD from hyperglycemia [17–19]. In individuals with the Hp2-2 phenotype (~ 40% worldwide [20]), the antioxidant abilities of the Hp protein are compromised during hyperglycemia compared to those with the non-Hp2-2 phenotypes(Hp1-1 and Hp2-1). This results in increased susceptibility to atherosclerosis, and eventually incident CAD (such as fatal and non-fatal myocardial infarction) 17, 21]. Consequently, targeting lower glycated hemoglobin (HbA_1c_) levels may be more effective in reducing CAD risk in people with the Hp2-2 phenotype and hyperglycemia, as people with the Hp2-2 phenotype are more susceptible to vascular damage from high HbA_1c_.

Our prior work in the Action to Control Cardiovascular Risk in Diabetes (ACCORD) [1] and the Action in Diabetes and Vascular Disease: Preterax and Diamicron MR Controlled Evaluation (ADVANCE) [2] studies showed that only a subset of people with the Hp2-2 phenotype benefitted from intensive glycemic therapy to achieve near euglycemia [14]. We also demonstrated that randomization to the Action for Health in Diabetes (Look AHEAD) [22] intensive lifestyle intervention (ILI) for weight loss (caloric restriction and increased physical activity) versus diabetes support and education (DSE) did not reduce CAD in either Hp phenotype group [23]. The null results from our re-analysis of the Look AHEAD data by Hp phenotype may be due to the intervention’s limited impact on glycemic control. In these studies, we analyzed the data based on intervention assignment, following the intention-to-treat principle; consistent with the original studies, discrepancies between target and actual HbA_1c_ levels were not considered in the analyses.

When we analyzed repeated measurements of HbA_1c_ in the ACCORD study by Hp phenotype, we found that attaining HbA_1c_ > 8.0% compared with 7.0-7.9% was associated with increased incident CAD risk among participants with only the Hp2-2 phenotype [14]. No similar association was observed among participants without the Hp2-2 phenotype. The earlier results in our ACCORD and ADVANCE intention-to-treat analyses may have been influenced by not having high HbA_1c_ levels (i.e., ≥ 8.0%) rather than by achieving intensive glycemic control in individuals with the Hp2-2 phenotype. We did not find evidence that having an HbA_1c_ concentration < 7.0% prevents CAD in individuals with or without the Hp2-2 phenotype.

The ACCORD and ADVANCE studies assessed pharmacological interventions to lower glucose and primarily included middle-aged and older White males at high risk of CVD with long-standing T2DM and hyperglycemia. Therefore, the ideal glycemic target for preventing CAD in individuals with different Hp phenotypes remains unclear, particularly for those with diverse demographic and clinical characteristics. The primary objective of the present study was to determine whether the relationship between attaining specific HbA_1c_ targets (< 6.5%, 6.5–6.9%, and ≥ 8.0% compared to 7.0-7.9%) over time and risk of incident CAD is dependent on Hp phenotype in the Look AHEAD study, which represents a different demographic and clinical population and intervention. We also assessed for heterogeneity of this relationship within subgroups decided upon *a priori*, including sex, age, race, diabetes duration, and previous CVD at baseline.

## Methods

### Study design and participants

We conducted a hypothesis-driven re-analysis of the original Look AHEAD trial to include Hp phenotype and repeated measurements of HbA_1c_ and covariables throughout the study’s duration (the median follow-up was 14.8 years). The design, methods, and major findings of the Look AHEAD trial (ClinicalTrials.gov identifier: NCT00017953) have been reported previously [22, 24]. Briefly, the Look AHEAD trial was a multi-centre randomized controlled trial that compared the effect of an ILI designed to achieve weight loss of at least 7% through caloric restriction and increased physical activity with a control regimen of DSE on CVD morbidity and mortality over time in 5,145 adults with T2DM. Participants were enrolled between 2001 and 2004 from 16 study centres in the United States [22]. To be eligible for enrollment, individuals must have been aged 45–76 years, and have had a diagnosis of T2DM (self-reported but verified by the use of glucose-lowering medication, a physician’s report, or glucose levels), a HbA_1c_ level of ≤ 11%, a body mass index (BMI) ≥ 25 kg/m2 (≥ 27 kg/m2 if using insulin), a diastolic blood pressure < 100 mmHg, a triglyceride level of < 600 mg/dL, the ability to complete a valid maximal exercise test, and an established relationship with a primary care provider [22]. Participants with and without a history of CVD were included. Of the 5,145 Look AHEAD participants who were enrolled, approximately 60% were female and 63%, 16%, 13%, and 5% identified as White, Black, Hispanic, and Native American or Alaskan Native, respectively.

The Look AHEAD intervention was terminated on September 14, 2012 (after a median follow-up of 9.6 years) based on futility for the primary outcome and recommendation from the data and safety monitoring board; however, participants continued to be followed with clinic visits until April 30, 2018. The Look AHEAD study protocol was approved by institutional review boards at each centre and all participants included in the present study provided written informed consent, including consent for future research.

### Haptoglobin phenotyping

Hp phenotyping was performed using a high throughput enzyme-linked immunosorbent assay (ELISA) method that distinguishes between Hp types (1–1, 2 − 1, and 2–2) by analyzing the size and structure variations of Hp proteins present in a person’s blood sample [15]. This method can distinguish the Hp2-2 protein from the non-Hp2-2 proteins with a sensitivity of 99% and a specificity of 98.1% [25], and has successfully been used to determine Hp phenotype in previous studies [15, 18, 26]. The Hp phenotype and Hp genotype have a direct 1:1 correspondence, and the Hp type remains constant in a person over time. Therefore, blood samples from any subsequent visits were used. Serum samples from 603 Look AHEAD participants were unavailable for Hp phenotyping either due to prior depletion for measuring other biomarkers or because these participants did not consent to sharing their data externally. Therefore, Hp phenotyping was completed for 4,542 (88.3%) Look AHEAD participants. In our earlier intention-to-treat analysis of Look AHEAD data, we employed inverse probability weighting in a sensitivity analysis to evaluate the potential selection bias arising from the exclusion of individuals with missing Hp phenotype data from all enrolled participants. The findings remained consistent, suggesting the absence of selection bias.

### Measurement of glycated hemoglobin and covariables

HbA_1c_ levels were measured at baseline, annually from years 1–4, and at years 6, 8, and 10 by the central biochemistry laboratory (Northwest Lipid Research Laboratories, University of Washington, Seattle, WA) using standard protocols [24]. Eleven participants were missing a baseline HbA_1c_ measurement or did not have any HbA_1c_ measurements recorded during the follow-up period and were excluded, leaving 4,531 participants available for the present analysis. We grouped the time dependent HbA_1c_ data into four clinically relevant categories: <6.5%, 6.5–6.9%, 7.0–7.9%, and ≥8.0%. These cut points were chosen because the relationship between the Hp2-2 phenotype and CAD risk is associated with the HbA_1c_ cut-point of ≥ 6.5% [18, 19], the current American Diabetes Association clinical practice guidelines recommend a less strict HbA_1c_ goal of <8.0% for individuals with a history of microvascular or macrovascular disease and long-standing diabetes [27], the target HbA_1c_ level for the control group in the ACCORD trial was 7.0-7.9% [28], and because our previous studies also used similar HbA_1c_ cut points when examining the relationship between time-varying attained HbA_1c_ and CAD in different Hp phenotype groups [14, 29].

Sociodemographic characteristics, lifestyle factors, and history of CVD were self-reported by participants at baseline. Repeated assessment of blood pressure and anthropometric measures occurred during annual study visits by certified masked staff using standardized protocols [24]. Participants provided their prescription medications at baseline and annual visits, and study staff recorded the medication names in the study database. The central biochemistry laboratory conducted laboratory measurements annually from baseline to year 4, and then every other year up to year 10.

### Outcomes

The primary outcome reported in our study was major CAD, which was defined as a composite of the following pre-specified Look AHEAD outcomes [22]: fatal and non-fatal MI, hospitalization for angina, and possible fatal CAD. Outcome events were validated by end-point adjudicators who were blinded to participants’ study group assignments. We decided to focus on studying CAD events as the primary outcome instead of the Look AHEAD study’s primary outcome, which included a composite of death from cardiovascular causes, nonfatal myocardial infarction, non-fatal stroke, or hospitalization for angina. This decision was made because stroke is more closely associated with the Hp1-1 phenotype rather than the Hp2-2 phenotype [30, 31] and can have etiology unrelated to atherosclerosis, indicating that CAD and stroke outcomes should be separated from a composite CVD outcome for analyses stratified by Hp phenotype. In sensitivity analyses, we examined HbA_1c_ targets in relation to other Look AHEAD outcomes, including the original study primary composite outcome of CVD (death from cardiovascular causes, non-fatal myocardial infarction, non-fatal stroke, or hospitalization for angina), death from any cause, and severe hypoglycemia events (loss of consciousness, seizure, or a glucose < 70 mg/dL that prevented self-treatment and required assistance of another person) [22].

### Statistical analysis

All analyses were conducted using Stata/SE statistical software version 18 (StataCorp, College Station, TX) according to an analysis plan developed *a priori*. All analyses were conducted at a 2-tailed α level of 0.05. For some analyses, we combined the Hp1-1 and Hp2-1 phenotypes to form a group of Hp1 allele carriers (referred to as the non-Hp 2–2 group). We made this decision to allow for easier comparison with other studies that have used the same approach [18, 34]. Our decision was influenced by the low frequency of the Hp1-1 phenotype, particularly for subgroup analyses, and because Hp1-1 and Hp2-1 both lack cyclic polymers, unlike Hp2-2 [35]. The smaller size of the Hp1-1 and Hp2-1 proteins allows them to penetrate environments that may restrict the larger, cyclic Hp2-2 protein, which can affect uptake of the Hp-hemoglobin complex by the CD163 receptor and oxidation by hemoglobin and heme release from the Hp-hemoglobin complex [35]. However, we also conducted the analysis separately for the Hp1-1 and Hp2-1 groups, although the sample size in the Hp1-1 group was potentially small for subgroup analyses.

We grouped participants based on a combination of Hp phenotype and HbA_1c_ categories, and summarized baseline characteristics, comparing the groups using 1-way analysis of variance (ANOVA) or Kruskal-Wallis tests for continuous variables and chi-square tests for categorical variables. T-tests were used to compare the mean HbA_1c_ levels at each time point among the Hp phenotype groups to determine if there were differences in mean HbA_1c_ levels over the study duration.

To estimate the long-term effect of HbA_1c_ targets (< 6.5%, 6.5–6.9%, and ≥ 8.0% compared to 7.0-7.9%) on risk of CAD in the non-Hp2-2 and Hp2-2 phenotype groups separately, we employed Cox proportional hazards models stratified by Hp phenotype group to estimate adjusted hazard ratios (aHR) and 95% confidence intervals (CI). Multivariable Cox models were adjusted for the time-independent variables using data for baseline age (years), sex (male, female), study site, self-reported race/ethnicity (White, Black, Hispanic, unspecified), education (high school or less, some college, college graduate, graduate school, other), income (USD; <$20,000, $20,000-$39,999, $60,000-$79,999, ≥$80,000, missing), study group assignment (ILI, DSE), history of CVD at baseline (yes, no), current smoking at baseline (yes, no), baseline alcohol consumption (yes, no), diabetes duration (≤ 10 years, > 10 years), and the time-dependent variables low-density lipoprotein cholesterol (mg/dL), BMI (kg/m^2^), diastolic blood pressure (mmHg), diabetes medications use (yes, no), anti-hypertensive medication use (yes, no), and lipid medication use (yes, no). Time-varying covariables were used to link the most recent measure of each variable to incident CAD at the time of an event, reducing potential within-person measurement error and misclassification bias from a single baseline measurement. Cluster variance estimates were used to account for within-subject correlation of repeated measures. Interactions between HbA_1c_ targets (included as a continuous variable) and Hp phenotype were tested by adding an interaction term to the model. The proportional hazards assumption was checked by including time-dependent covariables in the model and testing statistical significance. To handle missing data in the study, we used the last observation carried forward method for the following time-dependent variables: HbA_1c_ (1.3% missing), low-density lipoprotein (2.3% missing), BMI (0.7% missing), diastolic blood pressure (0.7% missing), diabetes medication use (1.9% missing), anti-hypertensive medication use (2.2% missing), and lipid medication use (3.1% missing). Participants were observed for up to 16 years and contributed follow-up time from the date of their first HbA_1c_ measurement until the date of documented outcome, death, or the end of the follow-up period (April 30, 2018), whichever came first.

Further stratified analyses by sex, age, race, diabetes duration, and previous CVD at baseline were performed for the primary CAD outcome in each Hp phenotype group separately. These subgroups were chosen *a priori* because current reporting guidelines recommend disaggregation of results by sex [36] and race [37], the frequency of the Hp2-2 phenotype differs among race-based and geographic populations [20], and current diabetes clinical practice guidelines suggest that diabetes duration and established CVD are important factors for glycemic control [10, 27]. Interactions between HbA_1c_ targets (continuous variable) and each subgroup were tested by adding an interaction term to the model for each Hp phenotype group.

To visualize the relationship between continuous HbA_1c_ and CAD in each Hp phenotype group across the range of HbA_1c_ in this study, we constructed restricted cubic spline models with knots placed at evenly spaced percentiles (5, 27.5, 50, 72.5 and 95) of HbA_1c_ [38]. The regression plot was truncated at the 5th and 95th percentiles, with the reference set at 7.0%.

## Results

The distribution of Hp phenotype frequencies was 19.7% Hp1-1 (*n* = 892), 45.3% Hp2-1 (*n* = 2,052), and 35.0% Hp2-2 (*n* = 1,587) (data not shown), which was not in Hardy-Weinberg equilibrium (HWE) (p-value < 0.01). Baseline characteristics are described according to Hp phenotype and HbA_1c_ categories (Table 1).

**Table 1:** Baseline characteristics of Look AHEAD study participants stratified by glycated hemoglobin (HbA_1c_)cut points at baseline and haptoglobin (Hp) phenotype group*

	Non-Hp2-2 Phenotypes (*n* = 2,944)					Hp2-2 Phenotype (*n* = 1,587)					
	HbA_1c_ (%)					HbA_1c_ (%)					
	< 6.5	6.5–6.9	7.0-7.9	**≥** 8.0	P-value	< 6.5	6.5–6.9	7.0-7.9	**≥** 8.0	P-value	Overall P-value^‡^
N	734 (24.9%)	637 (21.6%)	908 (30.8%)	665 (22.6%)		413 (26.0%)	322 (20.3%)	504 (31.8%)	348 (21.9%)		
Age (years)	59.0 ± 6.7	59.6 ± 6.7	59.5 ± 6.7	57.6 ± 6.7	< 0.01	59.6 ± 6.8	59.3 ± 6.4	58.8 ± 6.9	57.5 ± 6.3	< 0.01	0.54
Female sex	440 (59.9%)	386 (60.6%)	537 (59.1%)	403 (60.6%)	0.92	227 (55.0%)	180 (55.9%)	302 (59.9%)	195 (56.0%)	0.43	0.05
Married	494 (67.3%)	443 (69.7%)	632 (69.6%)	424 (63.8%)	0.06	301 (72.9%)	213 (66.1%)	358 (71.0%)	231 (66.4%)	0.11	0.22
Race					< 0.01					0.01	< 0.01
Black	125 (17.0%)	105 (16.5%)	182 (20.0%)	166 (25.0%)		33 (8.0%)	31 (9.6%)	53 (10.5%)	48 (13.8%)		
White	483 (65.8%)	435 (68.4%)	554 (61.0%)	358 (53.8%)		323 (78.2%)	251 (78.0%)	385 (76.4%)	237 (68.1%)		
Hispanic	97 (13.2%)	73 (11.5%)	141 (15.5%)	120 (18.0%)		37 (9.0%)	33 (10.2%)	44 (8.7%)	51 (14.7%)		
Unspecified	29 (4.0%)	23 (3.6%)	31 (3.4%)	21 (3.2%)		20 (4.8%)	7 (2.2%)	22 (4.4%)	12 (3.4%)		
History of CVD	77 (10.5%)	79 (12.4%)	151 (16.6%)	101 (15.2%)	< 0.01	49 (11.9%)	51 (15.8%)	63 (12.5%)	50 (14.4%)	0.37	0.68
Education					0.01					0.05	0.03
High school or less	126 (17.2%)	99 (15.5%)	193 (21.3%)	158 (23.8%)		66 (16.0%)	46 (14.3%)	81 (16.1%)	70 (20.1%)		
Some college	211 (28.8%)	194 (30.5%)	276 (30.4%)	174 (26.2%)		115 (27.8%)	82 (25.5%)	135 (26.8%)	111 (31.9%)		
College graduate	227 (31.0%)	191 (30.0%)	256 (28.2%)	213 (32.0%)		129 (31.2%)	113 (35.1%)	168 (33.3%)	99 (28.4%)		
Graduate school	148 (20.2%)	140 (22.0%)	162 (17.9%)	108 (16.2%)		101 (24.5%)	74 (23.0%)	105 (20.8%)	62 (17.8%)		
Other	21 (2.9%)	13 (2.0%)	20 (2.2%)	12 (1.8%)		2 (0.5%)	7 (2.2%)	15 (3.0%)	6 (1.7%)		
Income in the last 12 months (USD)					< 0.01					0.18	< 0.01
<20,000	66 (9.0%)	63 (9.9%)	86 (9.5%)	101 (15.2%)		31 (7.5%)	26 (8.1%)	35 (6.9%)	34 (9.8%)		
20,000–39,999	142 (19.3%)	101 (15.9%)	193 (21.3%)	131 (19.7%)		70 (16.9%)	69 (21.4%)	84 (16.7%)	65 (18.7%)		
40,000–59,999	149 (20.3%)	102 (16.0%)	171 (18.8%)	130 (19.5%)		79 (19.1%)	56 (17.4%)	92 (18.3%)	68 (19.5%)		
60,000–79,999	114 (15.5%)	114 (17.9%)	133 (14.6%)	92 (13.8%)		60 (14.5%)	40 (12.4%)	68 (13.5%)	63 (18.1%)		
** ≥**80,000	191 (26.0%)	173 (27.2%)	233 (25.7%)	167 (25.1%)		130 (31.5%)	97 (30.1%)	170 (33.7%)	99 (28.4%)		
Missing	72 (9.8%)	84 (13.2%)	92 (10.1%)	44 (6.6%)		43 (10.4%)	34 (10.6%)	55 (10.9%)	19 (5.5%)		
Smoking status					0.45					0.30	0.98
Never	361 (49.3%)	321 (50.5%)	447 (49.3%)	343 (51.7%)		201 (48.7%)	156 (48.6%)	266 (52.8%)	168 (48.6%)		
Past	348 (47.5%)	291 (45.8%)	423 (46.7%)	286 (43.1%)		202 (48.9%)	153 (47.7%)	215 (42.7%)	161 (46.5%)		
Current	23 (3.1%)	24 (3.8%)	36 (4.0%)	34 (5.1%)		10 (2.4%)	12 (3.7%)	23 (4.6%)	17 (4.9%)		
Consumed alcohol in the last year	462 (63.0%)	393 (61.7%)	541 (59.6%)	393 (59.1%)	0.37	291 (70.6%)	209 (64.9%)	321 (63.7%)	207 (59.7%)	0.02	0.01
Family history of diabetes	471 (64.2%)	411 (64.5%)	588 (64.8%)	455 (68.4%)	0.31	241 (58.4%)	207 (64.3%)	316 (62.7%)	237 (68.3%)	0.04	0.13
Family history of stroke	288 (39.2%)	246 (38.6%)	341 (37.6%)	239 (35.9%)	0.61	155 (37.5%)	110 (34.2%)	189 (37.5%)	139 (40.1%)	0.48	0.77
Family history of myocardial infarction	407 (55.4%)	342 (53.7%)	488 (53.7%)	348 (52.3%)	0.71	216 (52.3%)	170 (52.8%)	294 (58.3%)	189 (54.5%)	0.25	0.54
Diabetes severity^†^					< 0.01					< 0.01	0.98
1	175 (24.5%)	98 (15.8%)	80 (9.0%)	19 (3.0%)		90 (22.6%)	34 (11.3%)	60 (12.3%)	20 (6.0%)		
2	330 (46.2%)	313 (50.3%)	431 (48.6%)	288 (44.9%)		182 (45.7%)	157 (52.2%)	221 (45.3%)	160 (48.0%)		
3	146 (20.4%)	137 (22.0%)	189 (21.3%)	130 (20.2%)		98 (24.6%)	70 (23.3%)	103 (21.1%)	53 (15.9%)		
4	13 (1.8%)	18 (2.9%)	41 (4.6%)	43 (6.7%)		4 (1.0%)	10 (3.3%)	22 (4.5%)	22 (6.6%)		
5	28 (3.9%)	32 (5.1%)	78 (8.8%)	87 (13.6%)		17 (4.3%)	19 (6.3%)	47 (9.6%)	39 (11.7%)		
6	22 (3.1%)	24 (3.9%)	67 (7.6%)	75 (11.7%)		7 (1.8%)	11 (3.7%)	35 (7.2%)	39 (11.7%)		
Medications											
Insulin	34 (4.8%)	59 (9.6%)	156 (17.9%)	192 (29.5%)	< 0.01	19 (4.7%)	37 (12.1%)	92 (19.0%)	91 (26.7%)	< 0.01	0.92
Metformin	316 (43.9%)	334 (53.6%)	513 (58.2%)	380 (57.8%)	< 0.01	200 (49.1%)	188 (59.5%)	281 (56.9%)	197 (57.6%)	0.02	0.21
TZD	168 (23.5%)	161 (25.9%)	256 (29.0%)	205 (31.7%)	< 0.01	105 (26.1%)	81 (26.6%)	138 (28.4%)	92 (27.3%)	0.88	0.79
Any diabetes medication	547 (75.4%)	532 (84.3%)	818 (91.0%)	639 (96.5%)	< 0.01	313 (76.5%)	282 (88.1%)	439 (88.2%)	321 (92.8%)	< 0.01	0.45
Beta blocker	151 (20.6%)	131 (20.6%)	195 (21.5%)	161 (24.2%)	0.32	96 (23.2%)	78 (24.2%)	123 (24.4%)	71 (20.4%)	0.55	0.24
Angiotensin converting enzyme inhibitors	297 (41.2%)	260 (41.9%)	398 (45.1%)	302 (46.0%)	0.19	169 (41.8%)	156 (50.5%)	219 (45.1%)	149 (43.8%)	0.13	0.38
Diuretic	232 (32.4%)	192 (31.0%)	315 (35.7%)	207 (31.9%)	0.22	116 (28.6%)	96 (31.1%)	165 (33.9%)	92 (27.2%)	0.17	0.08
Any anti-hypertensive medication	519 (71.5%)	444 (70.6%)	672 (75.2%)	485 (73.4%)	0.19	297 (72.8%)	241 (76.3%)	365 (74.5%)	242 (70.6%)	0.37	0.62
Statin	295 (41.0%)	284 (45.6%)	425 (48.0%)	278 (42.7%)	0.03	195 (47.8%)	166 (53.2%)	248 (50.5%)	152 (44.7%)	0.15	< 0.01
Any lipid lowering medication	337 (46.7%)	310 (49.8%)	470 (53.0%)	306 (46.9%)	0.04	225 (55.1%)	181 (57.8%)	265 (54.0%)	168 (49.4%)	0.18	< 0.01
Anti-depressant	116 (16.2%)	96 (15.6%)	153 (17.5%)	111 (17.1%)	0.77	71 (17.6%)	54 (17.6%)	92 (18.9%)	68 (20.2%)	0.79	0.11
Weight (kg)	99.7 ± 18.8	100.6 ± 19.6	100.7 ± 18.8	102.7 ± 20.2	0.03	99.7 ± 18.9	102.5 ± 19.5	101.7 ± 19.0	102.3 ± 19.5	0.17	0.33
Body mass index (kg/m^2^)	35.6 ± 5.9	35.7 ± 6.2	36.1 ± 5.8	36.6 ± 6.0	0.01	35.0 ± 5.5	36.2 ± 6.0	36.2 ± 6.0	36.1 ± 5.8	< 0.01	0.55
Waist circumference (cm)	112.3 ± 14.1	113.0 ± 13.9	114.6 ± 13.9	115.2 ± 14.9	< 0.01	111.8 ± 13.6	114.9 ± 13.5	114.4 ± 13.7	115.0 ± 13.9	< 0.01	0.78
Systolic blood pressure (mmHg)	127.3 ± 17.1	128.3 ± 16.5	130.3 ± 17.8	131.3 ± 17.2	< 0.01	127.4 ± 16.3	128.7 ± 17.1	129.3 ± 17.3	129.7 ± 17.2	0.23	0.30
Diastolic blood pressure (mmHg)	69.4 ± 9.5	69.9 ± 9.4	70.1 ± 9.6	71.4 ± 10.0	< 0.01	69.8 ± 9.3	70.1 ± 9.6	70.3 ± 9.4	71.2 ± 9.6	0.20	0.59
HDL cholesterol (mg/dL)											0.26
Males	38.4 ± 10.2	38.0 ± 7.9	38.5 ± 9.0	36.9 ± 8.7	0.15	38.7 ± 8.9	38.1 ± 9.4	38.4 ± 8.3	36.8 ± 8.6	0.25	0.91
Females	49.6 ± 12.6	47.5 ± 11.9	46.3 ± 10.6	46.5 ± 13.2	< 0.01	48.1 ± 12.7	47.9 ± 11.8	46.1 ± 11.9	47.1 ± 12.1	0.21	0.59
LDL cholesterol (mg/dL)	111.7 ± 30.6	113.3 ± 33.3	110.3 ± 31.7	116.5 ± 34.8	< 0.01	111.5 ± 31.4	106.5 ± 29.8	112.3 ± 30.5	116.1 ± 33.1	< 0.01	0.34
Triglycerides (mg/dL)	140 (97–194)	155 (106–215)	153 (107–219)	170 (114–246)	< 0.01	140 (103–205)	158 (110–223)	164 (111–232)	177 (123–243)	< 0.01	0.04
Diabetes duration	3 (1–6)	4 (2–8)	6 (3–10)	7 (4–12)	< 0.01	3 (1–7)	4 (2–9)	6 (3–10)	6 (4–11)	< 0.01	0.61

BMI, body mass index; CVD, cardiovascular disease; HDL, high-density lipoprotein; LDL, low-density lipoprotein; MI, myocardial infarction; TZD, thiazolidinediones

*Values are either mean ± standard deviation or median and interquartile range

^†^Diabetes severity categories are as follows: 1 = no glucose-lowering medications; 2 = oral glucose-lowering drug (not TZD), no insulin; 3 = TZD (with or without other oral drugs), no insulin; 4 = insulin alone; 5 = insulin with any oral glucose-lowering drugs (not TZD); 6 = insulin plus TZD, with or without other oral drugs

^‡^P-value comparing baseline characteristics between Hp phenotypes

**Fig. 1:**
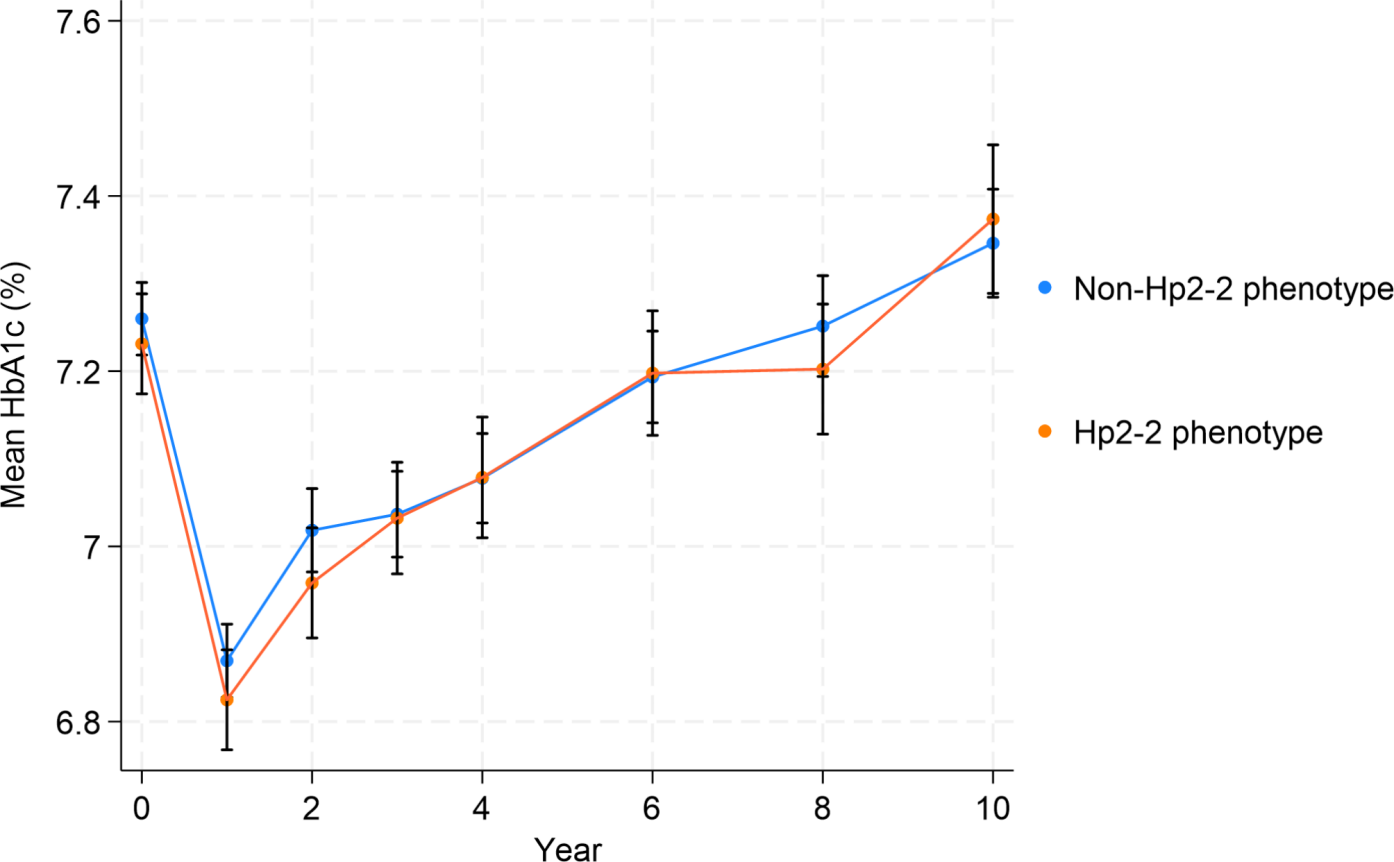
Mean HbA_1c_ levels over study duration by haptoglobin phenotype. Mean HbA_1c_ over study duration did not differ between haptoglobin phenotype groups in Look AHEAD participants. Unadjusted mean HbA_1c_ levels in participants measured at baseline and years 1–4, 6, 8, and 10 for Look AHEAD trial participants with the non-Hp2-2 phenotype (blue) and with the Hp2-2 phenotype (orange). Mean HbA_1c_ at each timepoint was compared between Hp phenotype groups using t-tests. The sample size for the non-Hp2-2 and Hp2-2 phenotypes at different time points was as follows: 2,944 and 1,587 at baseline, 2,835 and 1,541 at year 2, 2,795 and 1,501 at year 4, 2,729 and 1,469 at year 6, 2,597 and 1,392 at year 8, and 2,330 and 1,260 at year 10, respectively.

Baseline characteristics that differed between HbA_1c_ categories within the non-Hp2-2 phenotype group include age, race/ethnicity, history of CVD, education, income, diabetes severity, insulin use, metformin use, thiazolidinedione use, any diabetes medication use, statin use, any lipid-lowering medication use, weight, BMI, waist circumference, systolic and diastolic blood pressure, high-density lipoprotein (HDL) cholesterol (in females only), LDL cholesterol, triglycerides, and diabetes duration (Table 1). Within the Hp2-2 phenotype group, the baseline characteristics that differed between HbA_1c_ categories were age, race, alcohol consumption, family history of diabetes, diabetes severity, insulin use, metformin use, any diabetes medication use, BMI, waist circumference, LDL cholesterol, triglycerides, and diabetes duration. HbA_1c_ levels did not differ between Hp phenotype groups at any time point during the follow-up period (Fig. 1).

In multivariable adjusted Cox regression models, having HbA_1c_ < 6.5% compared with 7.0-7.9% was not significantly associated with a lower risk of CAD among participants with the Hp2-2 phenotype (aHR 0.80, 95% CI 0.57–1.12) but was among those with the non-Hp2-2 phenotype (0.71, 0.55–0.90 p, Hp phenotype*HbA_1c_ interaction = 0.79) (Table 2). In subgroup analyses, this association was present among participants with the non-Hp2-2 phenotype who were male (0.60, 0.44–0.83; p, subgroup*HbA_1c_ interaction = 0.29), who did not have a history of CVD at baseline (0.65, 0.47–0.90; p-interaction = 0.27), who were aged ≥ 65 years (0.64, 0.44–0.94; p-interaction = 0.66), who were White (0.68, 0.51–0.91; p-interaction = 0.38), and who had diabetes duration > 10 years at baseline (0.58, 0.35–0.95; p-interaction = 0.29). No associations were found between the other HbA_1c_ targets and CAD risk in participants with the non-Hp2-2 phenotype, regardless of whether they were grouped together or divided into subgroups. When the non-Hp2-2 phenotype group was divided into Hp1-1 and Hp2-1 phenotype groups, having HbA_1c_ levels below 6.5% compared to 7.0-7.9% was not significantly associated with a reduced risk of CAD in participants with the Hp1-1 phenotype (0.76, 0.49–1.18) (Table 2), although it was associated with a lower risk among those with the Hp2-1 phenotype (0.68, 0.50–0.92).

**Table 2:** Multivariable adjusted hazard ratios* for CAD^†^ events comparing having time-varying glycated hemoglobin (HbA_1c_) of < 6.5%, 6.5–6.9% and ≥ 8.0% to 7.0-7.9% in the Hp1-1, Hp2-1, Hp1-1/Hp2-1 combined, and Hp2-2 phenotype groups within the Look AHEAD trial using the last observation carried forward method for missing time-dependent variables

			HbA_1c_ (%)				
			< 6.5	6.5–6.9	7.0-7.9	≥ 8.0	
	No. of events	Person-days	aHR (95% CI)	aHR (95% CI)	Ref.	aHR (95% CI)	*P* value^‡^
**Hp1-1 Phenotype**							
Overall (*n* = 892)	146	4,265,327	0.76 (0.49–1.18)	0.65 (0.38–1.09)	Ref.	0.91 (0.59–1.39)	0.80
**By sex**							0.27
Male (*n* = 331)	82	1,483,196	0.63 (0.34–1.14)	0.46 (0.21-1.00)	Ref.	0.65 (0.38–1.11)	
Female (*n* = 561)	64	2,782,131	1.27 (0.60–2.67)	1.18 (0.52–2.66)	Ref.	1.61 (0.75–3.47)	
**By baseline CVD history**							0.83
No (*n* = 784)	102	3,852,279	0.83 (0.49–1.42)	0.83 (0.44–1.55)	Ref.	0.99 (0.59–1.68)	
Yes (*n* = 108)	44	413,048	1.23 (0.47–3.19)	0.37 (0.11–1.22)	Ref.	0.66 (0.28–1.56)	
**By age**							0.18
<65 years (*n* = 715)	105	3,491,835	0.65 (0.37–1.15)	0.61 (0.33–1.15)	Ref.	0.98 (0.61–1.57)	
≥65 years (*n* = 177)	41	773,492	1.20 (0.56–2.57)	0.79 (0.29–2.17)	Ref.	0.59 (0.22–1.57)	
**By race**							0.25
White (*n* = 448)	92	2,089,657	0.76 (0.45–1.31)	0.58 (0.31–1.10)	Ref.	0.71 (0.39–1.30)	
Black (*n* = 261)	36	1,262,611	1.63 (0.63–4.22)	0.58 (0.14–2.39)	Ref.	1.55 (0.72–3.33)	
Hispanic (*n* = 149)	14	747,050	0.17 (0.01–3.27)	1.84 (0.27–12.35)	Ref.	0.72 (0.18–2.79)	
**By diabetes duration**							0.96
≤10 years (*n* = 714)	103	3,466,047	0.75 (0.44–1.26)	0.64 (0.35–1.17)	Ref.	0.95 (0.54–1.66)	
>10 years (*n* = 175)	43	782,846	0.83 (0.37–1.84)	0.74 (0.23–2.39)	Ref.	0.94 (0.46–1.94)	
**Hp2-1 Phenotype**							
Overall (*n* = 2,052)	343	9,744,312	0.68 (0.50–0.92)	0.90 (0.66–1.24)	Ref.	1.26 (0.95–1.68)	0.80
**By sex**							0.78
Male (*n* = 847)	204	3,777,851	0.63 (0.42–0.93)	0.83 (0.56–1.25)	Ref.	1.16 (0.79–1.70)	
Female (*n* = 1,205)	139	5,966,461	0.75 (0.47–1.20)	1.00 (0.60–1.68)	Ref.	1.40 (0.89–2.21)	
**By baseline CVD history**							0.17
No (*n* = 1,752)	208	8,656,769	0.59 (0.39–0.88)	0.94 (0.63–1.41)	Ref.	1.23 (0.84–1.80)	
Yes (*n* = 300)	135	1,087,543	0.76 (0.49–1.16)	0.79 (0.48–1.30)	Ref.	1.19 (0.75–1.88)	
**By age**							0.74
<65 years (*n* = 1,606)	223	7,896,693	0.84 (0.57–1.26)	1.10 (0.72–1.66)	Ref.	1.46 (1.02–2.09)	
≥65 years (*n* = 446)	120	1,847,619	0.52 (0.33–0.82)	0.72 (0.44–1.18)	Ref.	0.78 (0.45–1.37)	
**By race**							0.06
White (*n* = 1,382)	258	6,448,548	0.65 (0.46–0.92)	0.84 (0.58–1.21)	Ref.	1.49 (1.08–2.06)	
Black (*n* = 317)	39	1,561,620	0.98 (0.40–2.38)	1.18 (0.41–3.40)	Ref.	1.24 (0.46–3.34)	
Hispanic (*n* = 282)	36	1,397,506	0.60 (0.21–1.73)	1.37 (0.54–3.49)	Ref.	0.53 (0.19–1.51)	
**By diabetes duration**							0.32
≤10 years (*n* = 1,635)	253	7,864,546	0.78 (0.54–1.11)	0.98 (0.67–1.42)	Ref.	1.57 (1.12–2.20)	
>10 years (*n* = 405)	87	1,829,922	0.52 (0.28–0.96)	0.87 (0.47–1.59)	Ref.	0.69 (0.39–1.21)	
**Hp1-1 and Hp2-1 Combined**							
Overall (*n* = 2,944)	489	14,009,639	0.71 (0.55–0.90)	0.83 (0.63–1.09)	Ref.	1.13 (0.89–1.43)	0.79
**By sex**							0.29
Male (*n* = 1,178)	286	5,261,047	0.60 (0.44–0.83)	0.71 (0.50–1.02)	Ref.	0.98 (0.72–1.34)	
Female (*n* = 1,766)	203	8,748,592	0.90 (0.61–1.34)	1.03 (0.68–1.59)	Ref.	1.32 (0.91–1.92)	
**By baseline CVD history**							0.27
No (*n* = 2,536)	310	12,509,048	0.65 (0.47–0.90)	0.90 (0.64–1.26)	Ref.	1.14 (0.84–1.55)	
Yes (*n* = 408)	179	1,500,591	0.78 (0.53–1.13)	0.67 (0.43–1.04)	Ref.	1.07 (0.73–1.56)	
**By age**							0.66
<65 years (*n* = 2,321)	328	11,388,528	0.75 (0.55–1.04)	0.92 (0.65–1.30)	Ref.	1.24 (0.93–1.65)	
≥65 years (*n* = 623)	161	2,621,111	0.64 (0.44–0.94)	0.71 (0.46–1.12)	Ref.	0.73 (0.45–1.16)	
**By race**							0.38
White (*n* = 1,830)	350	8,538,205	0.68 (0.51–0.91)	0.76 (0.55–1.04)	Ref.	1.24 (0.93–1.64)	
Black (*n* = 578)	75	2,824,231	1.08 (0.55–2.09)	0.84 (0.37–1.89)	Ref.	1.54 (0.83–2.84)	
Hispanic (*n* = 431)	50	2,144,556	0.45 (0.17–1.16)	1.29 (0.61–2.69)	Ref.	0.55 (0.26–1.20)	
**By diabetes duration**							0.29
≤10 years (*n* = 2,349)	356	11,330,593	0.76 (0.57–1.02)	0.87 (0.63–1.19)	Ref.	1.32 (0.99–1.76)	
>10 years (*n* = 580)	130	2,612,768	0.58 (0.35–0.95)	0.80 (0.48–1.34)	Ref.	0.77 (0.50–1.19)	
**Hp2-2 Phenotype**							
Overall (*n* = 1,587)	255	7,499,244	0.80 (0.57–1.12)	0.92 (0.65–1.32)	Ref.	1.06 (0.76–1.48)	0.79
**By sex**							0.53
Male (*n* = 683)	152	3,054,897	0.84 (0.55–1.29)	0.98 (0.61–1.56)	Ref.	1.03 (0.66–1.61)	
Female (*n* = 904)	103	4,444,347	0.76 (0.44–1.32)	0.84 (0.49–1.43)	Ref.	1.00 (0.59–1.71)	
**By baseline CVD history**							0.13
No (*n* = 1,374)	163	6,710,015	0.81 (0.54–1.23)	0.80 (0.51–1.25)	Ref.	0.83 (0.54–1.27)	
Yes (*n* = 213)	92	789,229	0.96 (0.51–1.81)	1.30 (0.70–2.41)	Ref.	1.79 (1.00-3.20)	
**By age**							0.80
<65 years (*n* = 1,272)	184	6,149,191	0.82 (0.55–1.22)	0.94 (0.62–1.42)	Ref.	0.95 (0.66–1.37)	
≥65 years (*n* = 315)	71	1,350,053	0.84 (0.44–1.61)	1.05 (0.52–2.13)	Ref.	1.39 (0.62–3.09)	
**By race**							0.84
White (*n* = 1,196)	203	5,596,269	0.79 (0.54–1.15)	0.83 (0.56–1.23)	Ref.	1.01 (0.70–1.46)	
Black (*n* = 165)	19	804,400	0.22 (0.03–1.64)	0.31 (0.10–1.03)	Ref.	0.63 (0.23–1.77)	
Hispanic (*n* = 165)	19	821,560	2.73 (0.63–11.80)	1.69 (0.29–9.89)	Ref.	2.24 (0.40-12.49)	
**By diabetes duration**							0.35
≤10 years (*n* = 1,255)	179	6,032,555	0.79 (0.53–1.18)	1.04 (0.69–1.57)	Ref.	1.04 (0.68–1.58)	
>10 years (*n* = 319)	74	1,403,911	0.82 (0.40–1.66)	0.58 (0.27–1.22)	Ref.	1.07 (0.59–1.92)	

aHR, adjusted hazard ratio; CAD, coronary artery disease; CI, confidence interval; CVD, cardiovascular disease; HbA_1c_, glycated hemoglobin; Hp, haptoglobin

*Models were adjusted for the time-independent variables age, sex, study site, race, education, income, study group assignment, history of CVD at baseline, baseline smoking status, baseline alcohol consumption, diabetes duration, and the time-dependent variables low-density lipoprotein cholesterol, body mass index, diastolic blood pressure, any diabetes medications, any anti-hypertensive medications, and any lipid medication, except for when stratified by one of these variables

^†^CAD is defined as a composite of the following pre-specified Look AHEAD outcomes: fatal and non-fatal myocardial infarction, hospitalization for angina, and possible fatal CAD

^‡^P-value for interaction between Hp phenotype and HbA_1c_ categories or between subgroup and HbA_1c_ categories. The p-value of 0.80 was obtained from an interaction test involving a three-level variable for Hp phenotype (Hp1-1, Hp2-1, Hp2-2). The p-value of 0.79 was obtained from an interaction test involving a two-level variable for Hp phenotype (Hp1-1 and Hp2-1 combined and Hp2-2)

Having HbA_1c_ ≥ 8.0% was associated with CAD risk among participants with the Hp2-2 phenotype who had a history of CVD at baseline (1.79, 1.00-3.20; p, subgroup*HbA_1c_ interaction = 0.13). No associations were observed between the other HbA_1c_ targets and the risk of CAD when participants with the Hp2-2 phenotype were grouped together or separated into subgroups.

**Fig. 2:**
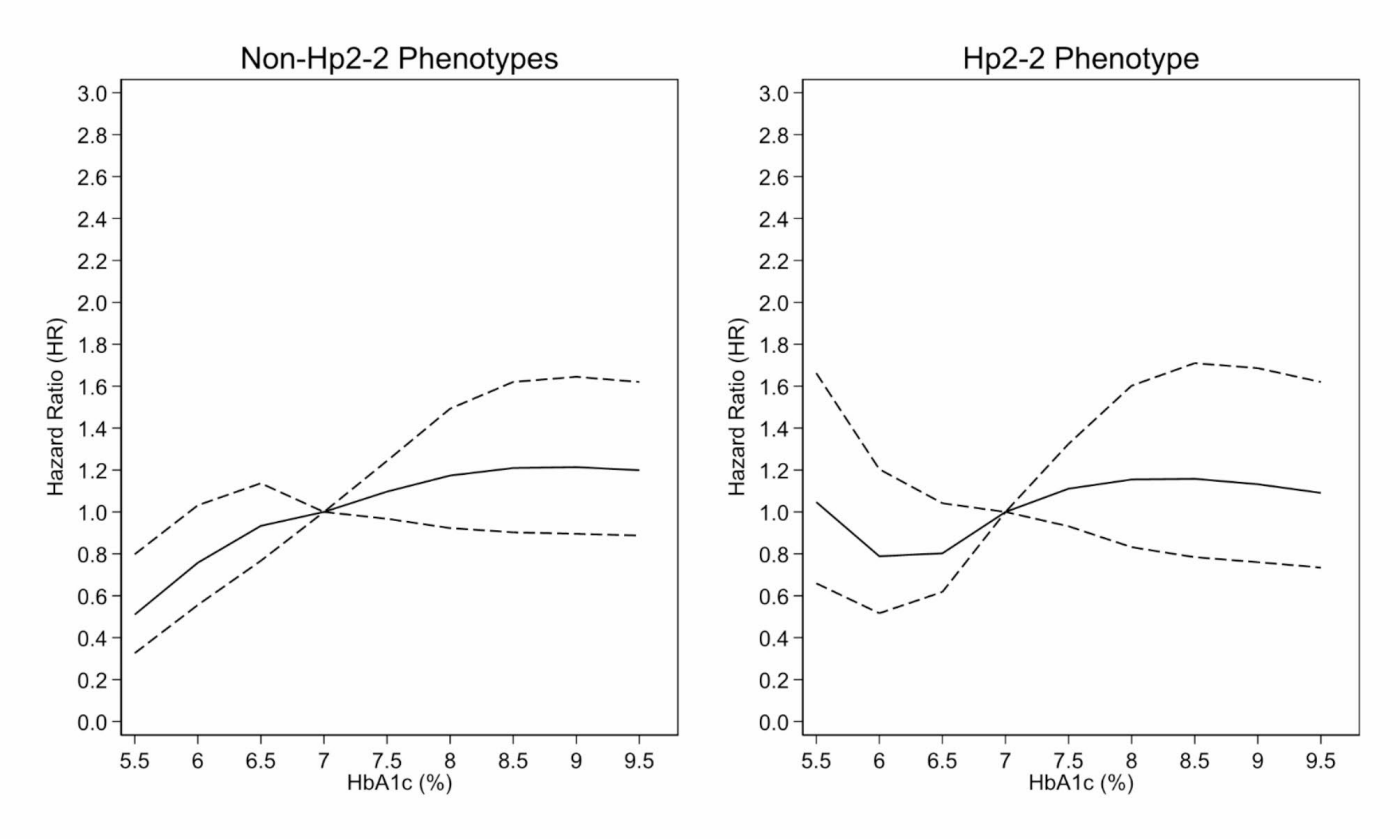
Association between HbA_1c_ and CAD in Look AHEAD participants by haptoglobin phenotype group. The solid line represents the fully adjusted hazard ratio, and the dotted lines represent the 95% confidence intervals. The plot was truncated at the 5th and 95th percentiles of HbA_1c_ (5.5% and 9.7% respectively).

Figure 2 displays the fully adjusted HR for CAD on the vertical axis, plotted against a range of continuous HbA_1c_ values using a restricted cubic spline model to account for non-linear relationships. In the non-Hp2-2 phenotype group, the risk of CAD increased as HbA_1c_ levels rose, reaching a plateau at HbA_1c_ > 8.0%. In the Hp2-2 phenotype group, a trend towards a U-shaped relationship was observed between HbA_1c_ levels and CAD risk within the range of 5.5–7.0%. The risk of CAD plateaued at HbA_1c_ values greater than 8.0%.

## Discussion

The results of this study, which analyzed HbA_1c_ measurements taken over a ten-year period from participants in the Look AHEAD study in relation to CAD stratified by Hp phenotype, are not consistent with the findings of our previous ACCORD [14] and Veterans Affairs Diabetes Trial (VADT) [29] studies of time-varying achieved HbA_1c_ and are unexpected. Our previous findings showed that achieving an HbA_1c_ level of < 7.0% was not associated with a decreased risk of CAD for either Hp phenotype group. In the present study, we found that individuals with the non-Hp2-2 phenotype and HbA_1c_ levels below 6.5% had a lower risk of CAD compared to those with HbA_1c_ levels between 7.0 and 7.9%. This association was observed within the Hp2-1 group after the non-Hp-2-2 phenotype group was split into Hp1-1 and Hp2-1 groups. However, this protective effect was not statistically significant in individuals with the Hp2-2 phenotype. In our analyses of the ACCORD trial, we found that having an HbA_1c_ level of 8.0% or higher, compared to 7.0-7.9%, was associated with a higher risk of CAD for the Hp2-2 phenotype only. In the VADT trial, having an HbA_1c_ level of 8.0% or higher was associated with a higher CAD risk among Hispanic participants with the non-Hp2-2 phenotype only [29]. In our current analysis, we observed that having an HbA_1c_ level of 8.0% or higher was associated with greater risk of CAD among Hp2-2 participants who had a prior history of CVD, but not in other subgroups of participants with the Hp2-2 phenotype or those with the non-Hp2-2 phenotype. The HbA_1c_ levels were consistent across both Hp phenotype groups during the Look AHEAD follow-up period, indicating that the results were not influenced by variations in HbA_1c_ levels among the different Hp phenotypes.

Our results are unexpected given the proposed biological mechanism linking Hp phenotype and CAD in hyperglycemia. In brief, individuals with the Hp2-2 phenotype produce a larger and less efficient Hp protein, which hinders the removal of free hemoglobin (Hb) from the blood (a primary function of Hp) compared to those with Hp1-1 and Hp2-1 phenotypes. In the presence of high blood glucose (HbA_1c_ ≥ 6.5%), the Hp2-2 function is further impaired [21], leading to a decreased ability of Hp to prevent oxidation of various lipid and protein substrates by free Hb [32, 39], as well as a reduced capacity of HDL to facilitate reverse cholesterol efflux. This ultimately results in plaque instability as Hp binds Hb to HDL [17, 21, 32, 40, 41]. Hp2-2 individuals have an increased amount of Hb on HDL, and the complex formed by glycated Hb and the Hp2-2 protein can act as a proatherogenic and proinflammatory compound [42]. This compound oxidatively modifies the HDL of Hp2-2 individuals with high blood glucose levels, leading to oxidative damage that heightens susceptibility to atherosclerosis, and ultimately CAD [17, 21, 32, 40, 41]. Given this biological mechanism, it would be expected that maintaining glycemic control would play a particularly important role in preventing CAD in individuals with the Hp2-2 phenotype, as it could potentially reduce Hp2-Hb-induced oxidative damage to blood vessels. This proposed mechanism is consistent with our previous ACCORD study, which demonstrated that individuals with the Hp2-2 phenotype and an HbA_1c_ level of ≥ 8.0% were at higher risk of CAD, while this association was not observed in individuals with non-Hp2-2 phenotypes.

Several factors, such as characteristics of the population studied and the sample size, may have influenced our results in the current study, making it challenging to draw definitive conclusions from the findings. Participants in the Look AHEAD study were required to have a BMI of at least 25.0 kg/m^2^ (or 27.0 kg/m^2^ if taking insulin) to be eligible for inclusion, whereas there was no BMI requirement for participation in the ACCORD and VADT trials. Consequently, participants in the Look AHEAD study had higher baseline BMIs (mean 36.0 kg/m^2^) compared to those in the other three studies (ACCORD: mean 32.2 kg/m^2^ and VADT: mean 31.3 kg/m^2^). In our previous intention-to-treat analysis of the Look AHEAD data, we found that there was a consistent difference in mean weight between the study groups throughout the follow-up period for the non-Hp2-2 phenotype group only [23]. There is evidence suggesting that Hp phenotype could impact the metabolic outcomes of weight loss interventions, which may have implications for the results of the present study. For example, two earlier studies conducted on individuals who were overweight or obese found greater improvements in clinical indicators of metabolic health, such as abdominal obesity (waist circumference, total body fat, and fat mass), plasma insulin levels, and insulin resistance, through either diet-induced weight loss or intermittent fasting in participants with the Hp1-1 phenotype compared to those with the Hp2-1 and Hp2-2 phenotypes [43]. Individuals with the Hp2-2 phenotype have exhibited inferior glycemic and insulinemic compensation compared to those with Hp1-1 and Hp2-1 phenotypes in the context of obesity [44]. Hp levels increase with greater adiposity [45], which could further exacerbate discrepancies between the phenotypes, especially since the Hp1-1 and Hp2-1 phenotypes are known to have higher antioxidant capabilities than the Hp2-2 phenotype [35]. Therefore, our observation of a protective effect of HbA_1c_ levels below 6.5% on CAD risk only among the non-Hp2-2 phenotype, and not the Hp2-2 phenotype, may be related to an unmeasured metabolic factor in this weight loss study.

Another possible explanation for the observed protective effect of HbA_1c_ levels below 6.5% on CAD in the non-Hp2-2 phenotype group, but not in the Hp2-2 phenotype group, could be insufficient statistical power in the Hp2-2 phenotype group. The non-Hp2-2 phenotype group had nearly twice as many participants and CAD cases as the Hp2-2 phenotype group, giving it greater statistical power to detect a significant effect. However, this explanation does not address the inconsistency with our ACCORD study, which found that having an HbA_1c_ level below 7.0% did not reduce CAD risk for both Hp phenotype groups.

The interaction terms we tested were not significant. However, it is important to note that testing interactions typically requires a larger sample size, 2 to 4 times more, to ensure adequate power compared to testing for main effects or subgroup effects [46, 47]. A previous study found high rates of false negative results in interaction tests when the treatment effect is significant in only one of the subgroups, as observed in our study [46]. Post hoc power calculations using the standard error and effect size from our study may make the interaction appear underpowered, rendering them meaningless.

Participants in the Look AHEAD trial may have had better metabolic health at the study’s outset due to their younger age, better physical fitness and baseline glycemic control, and less advanced diabetes compared to those in the ACCORD and VADT trials [1]. Participants in the Look AHEAD trial were required to have a baseline HbA_1c_ level of 11% or lower for eligibility [22], whereas there was no specified upper limit for HbA_1c_ levels for inclusion in the ACCORD [1] and VADT trials [3]. Baseline HbA_1c_ levels were indeed higher in these three trials compared to the Look AHEAD trial (ACCORD: median 8.1% and VADT: mean 9.4%, Look AHEAD: mean 7.3%). HbA_1c_ levels decreased in both the intervention and control groups during the initial year of the Look AHEAD trial, but then rose back to almost baseline levels in the following years. Conversely, HbA_1c_ levels stayed below baseline levels in the ACCORD and VADT trials over the course of the follow-up period. One potential reason for the discrepancy in our current findings compared to our previous results may be due to the limited variability in HbA_1c_ levels, particularly at higher values, which could have hindered the detection of an effect on CAD in the Hp2-2 phenotype group.

The Look AHEAD study participants had a shorter median duration of diabetes at baseline (5 years) and a lower percentage of participants with a prior cardiovascular event (14%) compared to participants from the other two studies. In contrast, the duration of diabetes was 10 years in ACCORD and 11.5 years in VADT, with a history of CVD reported in 35% of ACCORD participants and 40% of VADT participants. Therefore, participants in the other two studies had more advanced diabetes and were at higher cardiovascular risk at baseline compared to participants in the Look AHEAD study. In our *a priori*-defined subgroup analyses, we observed that individuals with the Hp2-2 phenotype and a history of CVD had a higher risk of CAD when their HbA_1c_ levels were ≥ 8.0% compared to 7.0-7.9%. It is possible that these middle-aged individuals with a history of CVD in the Look AHEAD trial had a degree of pre-existing cardiovascular damage (i.e., not permanent but requiring intervention) that could be improved by lowering HbA_1c_ levels.

The Hp phenotype frequencies in the Look AHEAD study participants were not in HWE overall. This deviation from HWE could be due to variations in Hp allele frequencies among different races/geographic regions [20], as the study included participants from diverse racial and ethnic backgrounds. When analyzed by race, the Hp phenotype frequencies were in HWE among White participants but not among Black participants (*p* < 0.01) (data not shown). This discrepancy may suggest selection bias; however, it is important to note that HWE assumptions are often not fully met in human populations [48, 49].

### Study strengths and limitations

Strengths of this study include a large sample of adults with T2DM who were overweight or obese, a large sample of females (who have historically been underrepresented in epidemiologic and clinical research on CAD [50, 51]), a long follow-up period, a prospective longitudinal design, and comprehensive repeated assessments of various anthropometric and laboratory measurements, as well as medication usage. By including time-dependent factors in our Cox regression models, we were able to minimize misclassification bias and address time-dependent confounding.

This study also had some limitations that should be acknowledged. First, our ability to detect significant associations in some subgroups in this study was limited by power due to small sample size and small numbers of events. Within the separate phenotypes, some subgroups had small sample sizes which could have resulted in either null results or spurious associations between HbA_1c_ and CAD in those subgroups, making it difficult to draw meaningful conclusions. Nevertheless, our findings are hypothesis-generating and could be used in a future meta-analysis to address our research question across various subgroups. Second, we cannot dismiss the possibility of residual confounding of the relationship between HbA_1c_ and the outcomes due to unknown or unmeasured factors such as HDL quality and function and health-related behaviours. Third, all participants included in this study were enrolled in an ILI trial, had a HbA_1c_ level of 11% or less at baseline, and were overweight or obese. Therefore, the findings may not be generalizable to populations with a wider range of HbA_1c_ levels, individuals with suboptimal T2DM management, or individuals of different body weights or metabolic risk profiles.

### Future directions

Additional prospective longitudinal studies are needed to confirm the results of this study, with an emphasis on including a more diverse range of participants in terms of age, sex, gender, race, ethnicity, and other clinical characteristics across various countries. Future studies should consider using more recent samples than those from the Look AHEAD, ACCORD, and VADT trials, as newer classes of medications have become available for managing T2DM (e.g., glucagon-like peptide-1 receptor agonists and sodium-glucose co-transporter-2 inhibitors) [52], potentially impacting the relationship between HbA_1c_ and CAD in the different Hp phenotype groups.

## Conclusions

In summary, our finding that HbA_1c_ levels below 6.5% compared to 7.0-7.9% was associated with a reduced risk of CAD in the non-Hp2-2 phenotype group does not align with the finding from our previous ACCORD study, where no CAD benefit was observed with HbA_1c_ below 7.0% in either Hp phenotype group. We also found that high HbA_1c_ levels (8.0% or higher) compared to 7.0-7.9% were associated with a greater risk of CAD only in individuals with the Hp2-2 phenotype and a previous history of CVD. These discrepancies may be due to differences in the study populations, intervention, and factors related to weight loss. We also cannot dismiss the possibility of residual confounding due to unknown or unmeasured external or lifestyle factors, such as eating disorders, time-restricted eating, and untreated depression. Additional research is required to establish the conclusiveness of these results.

## Electronic supplementary material

Below is the link to the electronic supplementary material.

Supplementary Material 1

## Data Availability

The Look AHEAD datasets used in the current study can be accessed from the National Institute of Diabetes and Digestive and Kidney Diseases Central Repository, https://repository.niddk.nih.gov/studies/look-ahead/. The haptoglobin phenotype dataset generated from this study is available upon reasonable request from the corresponding author.

## Abbreviations

ACCORD: Action to Control Cardiovascular Risk in Diabetes
ADVANCE: Action in Diabetes and Vascular Disease: Preterax and Diamicron Modified Release Controlled Evaluation
aHR: Adjusted hazard ratio
BMI: Body mass index
CAD: Coronary artery disease
CI: Confidence interval
CVD: Cardiovascular disease
DSE: Diabetes support and education
ELISA: Enzyme linked immunosorbent assay
Hb: Hemoglobin
HbA_1c_: Glycated hemoglobin
HDL: High-density lipoprotein
Hp: Haptoglobin
HR: Hazard ratio
HWE: Hardy Weinberg Equilibrium
ILI: Intensive lifestyle intervention
Look AHEAD: Action for Health in Diabetes
VADT: Veterans Affairs Diabetes Trial
