# Supplementary material for: The relationship between repeated measurements of HbA1c and risk of coronary events among the common haptoglobin phenotype groups: the Action for Health in Diabetes (Look AHEAD) study
Source: Cardiovasc Diabetol. 2024 Oct 9;23:356. doi: 10.1186/s12933-024-02448-z (PMC11466022; doi:10.1186/s12933-024-02448-z)
Supplement: Supplementary file 1 — Supplementary Material 1 [file 12933_2024_2448_MOESM1_ESM.docx]

**Supplementary Table 1.** Multivariable-adjusted hazard ratios* for CVD, severe hypoglycemia, and total mortality, comparing having time-varying glycated hemoglobin (HbA_1c_) of <6.5%, 6.5-6.9% and ≥8.0% to having HbA_1c_ of 7.0-7.9% in the non-Hp2-2 and Hp2-2 phenotype groups within the Action for Health in Diabetes (Look AHEAD) trial using the last observation carried forward method.

|  |  | HbA_1c_ (%) | | | | |
| --- | --- | --- | --- | --- | --- | --- |
|  |  | *<6.5* | *6.5-6.9* | *7.0-7.9* | *≥8.0* |  |
|  | No. of events | aHR (95% CI) | aHR (95% CI) | Ref. | aHR (95% CI) | *P* value^†^ |
| **Non-Hp2-2 Phenotype (n=2,944)** |  |  |  |  |  |  |
| CVD | 597 | 0.74 (0.59-0.92) | 0.90 (0.70-1.15) | Ref. | 1.24 (0.99-1.54) | 0.78 |
| Severe hypoglycemia | 169 | 1.13 (0.75-1.70) | 0.84 (0.52-1.38) | Ref. | 1.27 (0.85-1.91) | 0.03 |
| Total mortality | 477 | 1.11 (0.86-1.42) | 0.93 (0.69-1.25) | Ref. | 1.40 (1.08-1.81) | 0.88 |
| **Hp2-2 Phenotype (n=1,587)** |  |  |  |  |  |  |
| CVD | 316 | 0.77 (0.57-1.04) | 0.89 (0.65-1.23) | Ref. | 1.10 (0.82-1.48) | 0.78 |
| Severe hypoglycemia | 74 | 0.55 (0.28-1.08) | 0.49 (0.22-1.12) | Ref. | 1.51 (0.85-2.66) | 0.03 |
| Total mortality | 250 | 1.06 (0.75-1.50) | 0.73 (0.48-1.12) | Ref. | 1.13 (0.79-1.61) | 0.88 |

aHR, adjusted hazard ratio; CI, confidence interval; CVD, cardiovascular disease; Hp, haptoglobin phenotype.

*Models were adjusted for the time-independent variables age, sex, study site, race, education, income, study group assignment, history of CVD at baseline, baseline smoking status, baseline alcohol consumption, diabetes duration, and the time-dependent variables low-density lipoprotein cholesterol, body mass index, diastolic blood pressure, any diabetes medications, any anti-hypertensive medications, and any lipid medication.

^†^P-value for interaction between Hp phenotype and HbA_1c_ categories.

**Supplementary Table 2.** Multivariable adjusted hazard ratios* for CAD† events comparing having time-varying glycated hemoglobin (HbA_1c_) of <6.5%, 6.5-6.9% and ≥8.0% to 7.0-7.9% in the non-Hp2-2 and Hp2-2 phenotype groups within the Action for Health in Diabetes (Look AHEAD) trial using the multiple imputation method.

|  | |  | **HbA_1c_ (%)** | | | |  |
| --- | --- | --- | --- | --- | --- | --- | --- |
|  |  |  | *<6.5* | *6.5-6.9* | *7.0-7.9* | *≥8.0* |  |
|  | No. of events | Person-days | aHR (95% CI) | aHR (95% CI) | Ref. | aHR (95% CI) | *P* value‡ |
| **Non-Hp2-2 Phenotype** |  |  |  |  |  |  |  |
| Overall (n=2,949) | 489 | 14,038,068 | 0.70 (0.55-0.90) | 0.81 (0.62-1.07) | Ref. | 1.11 (0.88-1.41) | 0.81 |
| **By sex** |  |  |  |  |  |  | 0.21 |
| Male (n=1,179) | 286 | 5,266,884 | 0.59 (0.43-0.81) | 0.69 (0.48-0.98) | Ref. | 0.96 (0.70-1.31) |  |
| Female (n=1,770) | 203 | 8,771,184 | 0.93 (0.63-1.37) | 1.05 (0.68-1.63) | Ref. | 1.34 (0.92-1.95) |  |
| **By baseline CVD history** |  |  |  |  |  |  | 0.34 |
| No (n=2,541) | 310 | 12,537,477 | 0.68 (0.50-0.94) | 0.88 (0.63-1.25) | Ref. | 1.17 (0.86-1.59) |  |
| Yes (n=408) | 179 | 1,500,591 | 0.74 (0.50-1.07) | 0.67 (0.42-1.06) | Ref. | 1.05 (0.71-1.53) |  |
| **By age** |  |  |  |  |  |  | 0.79 |
| <65 years (n=2,325) | 328 | 11,411,238 | 0.76 (0.56-1.05) | 0.90 (0.64-1.27) | Ref. | 1.21 (0.91-1.61) |  |
| ≥65 years (n=624) | 161 | 2,626,830 | 0.61 (0.41-0.91) | 0.69 (0.44-1.08) | Ref. | 0.73 (0.46-1.17) |  |
| **By race** |  |  |  |  |  |  | 0.48 |
| White (n=1,833) | 350 | 8,555,029 | 0.68 (0.51-0.91) | 0.74 (0.53-1.03) | Ref. | 1.23 (0.93-1.63) |  |
| Black (n=580) | 75 | 2,835,836 | 1.09 (0.57-2.08) | 0.76 (0.34-1.68) | Ref. | 1.43 (0.78-2.63) |  |
| Hispanic (n=431) | 50 | 2,144,556 | 0.52 (0.20-1.35) | 1.61 (0.80-3.25) | Ref. | 0.61 (0.28-1.32) |  |
| **By diabetes duration** |  |  |  |  |  |  | 0.36 |
| ≤10 years (n=2,353) | 356 | 11,353,185 | 0.75 (0.56-1.01) | 0.86 (0.62-1.18) | Ref. | 1.29 (0.97-1.72) |  |
| >10 years (n=581) | 130 | 2,618,605 | 0.58 (0.35-0.96) | 0.76 (0.44-1.32) | Ref. | 0.76 (0.49-1.17) |  |
| **Hp2-2 Phenotype** |  |  |  |  |  |  |  |
| Overall (n=1,590) | 255 | 7,515,262 | 0.80 (0.57-1.14) | 0.92 (0.65-1.32) | Ref. | 1.11 (0.79-1.54) | 0.81 |
| **By sex** |  |  |  |  |  |  | 0.59 |
| Male (n=684) | 152 | 3,059,806 | 0.87 (0.56-1.34) | 0.95 (0.61-1.50) | Ref. | 1.09 (0.70-1.68) |  |
| Female (n=906) | 103 | 4,455,456 | 0.75 (0.42-1.34) | 0.84 (0.49-1.46) | Ref. | 1.05 (0.62-1.77) |  |
| **By baseline CVD history** |  |  |  |  |  |  | 0.09 |
| No (n=1,377) | 163 | 6,726,033 | 0.83 (0.54-1.26) | 0.83 (0.53-1.32) | Ref. | 0.85 (0.55-1.32) |  |
| Yes (n=213) | 92 | 789,229 | 0.86 (0.45-1.64) | 1.15 (0.64-2.07) | Ref. | 1.71 (0.97-3.01) |  |
| **By age** |  |  |  |  |  |  | 0.82 |
| <65 years (n=1,275) | 184 | 6,165,209 | 0.83 (0.55-1.26) | 0.99 (0.65-1.50) | Ref. | 1.03 (0.72-1.48) |  |
| ≥65 years (n=315) | 71 | 1,350,053 | 0.86 (0.45-1.66) | 0.94 (0.46-1.94) | Ref. | 1.30 (0.56-3.04) |  |
| **By race** |  |  |  |  |  |  | 0.81 |
| White (n=1,197) | 203 | 5,601,178 | 0.80 (0.54-1.17) | 0.81 (0.54-1.21) | Ref. | 1.06 (0.73-1.54) |  |
| Black (n=167) | 19 | 815,509 | 0.16 (0.02-1.75) | 0.29 (0.09-0.97) | Ref. | 0.58 (0.19-1.78) |  |
| Hispanic (n=165) | 19 | 821,560 | 2.33 (0.48-11.28) | 1.76 (0.30-10.14) | Ref. | 2.22 (0.37-13.50) |  |
| **By diabetes duration** |  |  |  |  |  |  | 0.16 |
| ≤10 years (n=1,258) | 179 | 6,048,573 | 0.80 (0.53-1.22) | 1.06 (0.70-1.61) | Ref. | 1.01 (0.66-1.55) |  |
| >10 years (n=319) | 74 | 1,403,911 | 0.80 (0.39-1.67) | 0.55 (0.25-1.18) | Ref. | 1.22 (0.66-2.25) |  |

aHR, adjusted hazard ratio; CAD, coronary artery disease; CI, confidence interval; CVD, cardiovascular disease; HbA_1c_, glycated hemoglobin; Hp, haptoglobin

*Models were adjusted for the time-independent variables age, sex, study site, race, education, income, study group assignment, history of CVD at baseline, baseline smoking status, baseline alcohol consumption, diabetes duration, and the time-dependent variables low-density lipoprotein cholesterol, body mass index, diastolic blood pressure, any diabetes medications, any anti-hypertensive medications, and any lipid medication, except for when stratified by one of these variables.

_†_CAD is defined as a composite of the following pre-specified Look AHEAD outcomes: fatal and non-fatal myocardial infarction, hospitalization for angina, and possible fatal CAD.

_‡_P-value for interaction between Hp phenotype and HbA_1c_ categories or between subgroup and HbA_1c_ categories.

**Supplementary Table 3.** Multivariable-adjusted hazard ratios* for CVD, severe hypoglycemia, and total mortality, comparing having time-varying glycated hemoglobin (HbA_1c_) of <6.5%, 6.5-6.9% and ≥8.0% to having HbA_1c_ of 7.0-7.9% in the non-Hp2-2 and Hp2-2 phenotype groups within the Action for Health in Diabetes (Look AHEAD) trial using the multiple imputation method.

|  |  | HbA_1c_ (%) | | | | |
| --- | --- | --- | --- | --- | --- | --- |
|  |  | *<6.5* | *6.5-6.9* | *7.0-7.9* | *≥8.0* |  |
|  | No. of events | aHR (95% CI) | aHR (95% CI) | Ref. | aHR (95% CI) | *P* value^†^ |
| **Non-Hp2-2 Phenotype (n=2,949)** |  |  |  |  |  |  |
| CVD | 597 | 0.73 (0.58-0.91) | 0.90 (0.70-1.15) | Ref. | 1.24 (1.00-1.54) | 0.84 |
| Severe hypoglycemia | 169 | 1.10 (0.73-1.65) | 0.83 (0.50-1.36) | Ref. | 1.27 (0.85-1.90) | 0.04 |
| Total mortality | 477 | 1.09 (0.85-1.41) | 0.90 (0.66-1.22) | Ref. | 1.45 (1.11-1.88) | 0.98 |
| **Hp2-2 Phenotype (n=1,590)** |  |  |  |  |  |  |
| CVD | 316 | 0.78 (0.57-1.06) | 0.90 (0.65-1.24) | Ref. | 1.17 (0.87-1.57) | 0.84 |
| Severe hypoglycemia | 74 | 0.54 (0.27-1.10) | 0.54 (0.25-1.19) | Ref. | 1.54 (0.88-2.70) | 0.04 |
| Total mortality | 250 | 1.04 (0.73-1.47) | 0.73 (0.48-1.12) | Ref. | 1.12 (0.78-1.60) | 0.98 |

aHR, adjusted hazard ratio; CI, confidence interval; CVD, cardiovascular disease; Hp, haptoglobin phenotype.

*Models were adjusted for the time-independent variables age, sex, study site, race, education, income, study group assignment, history of CVD at baseline, baseline smoking status, baseline alcohol consumption, diabetes duration, and the time-dependent variables low-density lipoprotein cholesterol, body mass index, diastolic blood pressure, any diabetes medications, any anti-hypertensive medications, and any lipid medication.

^†^P-value for interaction between Hp phenotype and HbA_1c_ categories.

**Supplementary Table 4.** Multivariable adjusted hazard ratios for CAD* events comparing having time-varying glycated hemoglobin (HbA_1c_) of <6.5%, 6.5-6.9% and ≥8.0% to 7.0-7.9% in the non-Hp2-2 and Hp2-2 phenotype groups within the Action for Health in Diabetes (Look AHEAD) trial using progressive modelling.

|  | **HbA_1c_ (%)** | | | |  |
| --- | --- | --- | --- | --- | --- |
|  | *<6.5* | *6.5-6.9* | *7.0-7.9* | *≥8.0* |  |
|  | aHR (95% CI) | aHR (95% CI) | Ref. | aHR (95% CI) | *P* value§ |
| **Non-Hp2-2 Phenotype** |  |  |  |  |  |
| CAD cases, n | 127 | 82 | 145 | 135 |  |
| Person-days | 4,798,746 | 2,544,842 | 3,507,848 | 3,158,203 |  |
| Adjusted for age and sex | 0.60 (0.47-0.76) | 0.74 (0.56-0.96) | Ref. | 1.19 (0.94-1.50) | 0.63 |
| + Demographic and lifestyle factors† | 0.60 (0.47-0.76) | 0.76 (0.58-0.99) | Ref. | 1.20 (0.95-1.51) | 0.59 |
| + Clinical factors‡ | 0.71 (0.55-0.90) | 0.83 (0.63-1.09) | Ref. | 1.13 (0.89-1.43) | 0.79 |
| **Hp2-2 Phenotype** |  |  |  |  |  |
| CAD cases, n | 68 | 50 | 73 | 64 |  |
| Person-days | 2,509,075 | 1,472,516 | 1,935,940 | 1,581,713 |  |
| Adjusted for age and sex | 0.66 (0.47-0.91) | 0.85 (0.60-1.21) | Ref. | 1.18 (0.85-1.64) | 0.63 |
| + Demographic and lifestyle factors† | 0.69 (0.49-0.96) | 0.87 (0.61-1.24) | Ref. | 1.17 (0.84-1.63) | 0.59 |
| + Clinical factors‡ | 0.80 (0.57-1.12) | 0.92 (0.65-1.32) | Ref. | 1.06 (0.76-1.48) | 0.79 |

aHR, adjusted hazard ratio; CAD, coronary artery disease; CI, confidence interval; CVD, cardiovascular disease; HbA_1c_, glycated hemoglobin; Hp, haptoglobin

*CAD is defined as a composite of the following pre-specified Look AHEAD outcomes: fatal and non-fatal myocardial infarction, hospitalization for angina, and possible fatal CAD.

_†_In addition to age and sex, this model is further adjusted for demographic and lifestyle factors: study site, race, education, income, baseline smoking status, baseline alcohol consumption. These variables are time independent.

‡In addition to age, sex, demographic, and lifestyle factors, this model is adjusted for clinical factors: history of CVD (time independent), diabetes duration (time independent), and the time-dependent variables low-density lipoprotein cholesterol, body mass index, diastolic blood pressure, any diabetes medications, any anti-hypertensive medications, and any lipid medication.

§P-value for interaction between Hp phenotype and HbA_1c_ categories.

**Supplementary Table 5.** Multivariable adjusted hazard ratios* for CAD† events comparing having time-varying glycated hemoglobin (HbA_1c_) of 6.5-6.9%, 7.0-7.9%, and ≥8.0% to <6.5% in the non-Hp2-2 and Hp2-2 phenotype groups within the Action for Health in Diabetes (Look AHEAD) trial using the last observation carried forward method for missing time-dependent variables.

|  | |  | **HbA_1c_ (%)** | | | |  |
| --- | --- | --- | --- | --- | --- | --- | --- |
|  |  |  | *<6.5* | *6.5-6.9* | *7.0-7.9* | *≥8.0* |  |
|  | No. of events | Person-days | Ref. | aHR (95% CI) | aHR (95% CI) | aHR (95% CI) | *P* value‡ |
| **Non-Hp2-2 Phenotype** |  |  |  |  |  |  |  |
| Overall (n=2,944) | 489 | 14,009,639 | Ref. | 1.17 (0.89-1.56) | 1.41 (1.11-1.81) | 1.59 (1.22-2.07) | 0.79 |
| **By sex** |  |  |  |  |  |  | 0.29 |
| Male (n=1,178) | 286 | 5,261,047 | Ref. | 1.18 (0.81-1.72) | 1.66 (1.20-2.28) | 1.63 (1.13-2.34) |  |
| Female (n=1,766) | 203 | 8,748,592 | Ref. | 1.14 (0.75-1.76) | 1.11 (0.75-1.64) | 1.46 (0.98-2.16) |  |
| **By baseline CVD history** |  |  |  |  |  |  | 0.27 |
| No (n=2,536) | 310 | 12,509,048 | Ref. | 1.37 (0.96-1.96) | 1.53 (1.11-2.12) | 1.75 (1.24-2.45) |  |
| Yes (n=408) | 179 | 1,500,591 | Ref. | 0.86 (0.54-1.36) | 1.29 (0.88-1.87) | 1.38 (0.90-2.10) |  |
| **By age** |  |  |  |  |  |  | 0.66 |
| <65 years (n=2,321) | 328 | 11,388,528 | Ref. | 1.23 (0.85-1.77) | 1.33 (0.96-1.83) | 1.65 (1.19-2.27) |  |
| ≥65 years (n=623) | 161 | 2,621,111 | Ref. | 1.11 (0.72-1.72) | 1.56 (1.06-2.27) | 1.13 (0.68-1.86) |  |
| **By race** |  |  |  |  |  |  | 0.38 |
| White (n=1,830) | 350 | 8,538,205 | Ref. | 1.11 (0.80-1.54) | 1.46 (1.10-1.95) | 1.81 (1.32-2.47) |  |
| Black (n=578) | 75 | 2,824,231 | Ref. | 0.78 (0.35-1.73) | 0.93 (0.48-1.80) | 1.43 (0.78-2.60) |  |
| Hispanic (n=431) | 50 | 2,144,556 | Ref. | 2.86 (1.03-7.98) | 2.23 (0.86-5.73) | 1.23 (0.42-3.64) |  |
| **By diabetes duration** |  |  |  |  |  |  | 0.29 |
| ≤10 years (n=2,349) | 356 | 11,330,593 | Ref. | 1.13 (0.82-1.56) | 1.31 (0.98-1.75) | 1.73 (1.28-2.34) |  |
| >10 years (n=580) | 130 | 2,612,768 | Ref. | 1.38 (0.76-2.49) | 1.72 (1.05-2.84) | 1.33 (0.78-2.26) |  |
| **Hp2-2 Phenotype** |  |  |  |  |  |  |  |
| Overall (n=1,587) | 255 | 7,499,244 | Ref. | 1.16 (0.80-1.67) | 1.25 (0.89-1.76) | 1.33 (0.92-1.91) | 0.79 |
| **By sex** |  |  |  |  |  |  | 0.53 |
| Male (n=683) | 152 | 3,054,897 | Ref. | 1.16 (0.72-1.87) | 1.19 (0.78-1.83) | 1.23 (0.77-1.96) |  |
| Female (n=904) | 103 | 4,444,347 | Ref. | 1.11 (0.63-1.96) | 1.32 (0.76-2.29) | 1.32 (0.73-2.39) |  |
| **By baseline CVD history** |  |  |  |  |  |  | 0.13 |
| No (n=1,374) | 163 | 6,710,015 | Ref. | 0.98 (0.62-1.54) | 1.23 (0.81-1.86) | 1.01 (0.64-1.62) |  |
| Yes (n=213) | 92 | 789,229 | Ref. | 1.35 (0.71-2.54) | 1.04 (0.55-1.96) | 1.86 (0.96-3.60) |  |
| **By age** |  |  |  |  |  |  | 0.80 |
| <65 years (n=1,272) | 184 | 6,149,191 | Ref. | 1.15 (0.73-1.81) | 1.23 (0.82-1.83) | 1.17 (0.78-1.76) |  |
| ≥65 years (n=315) | 71 | 1,350,053 | Ref. | 1.25 (0.68-2.29) | 1.19 (0.62-2.27) | 1.65 (0.79-3.45) |  |
| **By race** |  |  |  |  |  |  | 0.84 |
| White (n=1,196) | 203 | 5,596,269 | Ref. | 1.05 (0.70-1.59) | 1.27 (0.87-1.86) | 1.28 (0.85-1.94) |  |
| Black (n=165) | 19 | 804,400 | Ref. | 1.43 (0.16-12.68) | 4.55 (0.61-33.86) | 2.89 (0.49-17.05) |  |
| Hispanic (n=165) | 19 | 821,560 | Ref. | 0.62 (0.16-2.45) | 0.37 (0.08-1.58) | 0.82 (0.32-2.11) |  |
| **By diabetes duration** |  |  |  |  |  |  | 0.35 |
| ≤10 years (n=1,255) | 179 | 6,032,555 | Ref. | 1.32 (0.87-1.99) | 1.26 (0.84-1.89) | 1.31 (0.83-2.06) |  |
| >10 years (n=319) | 74 | 1,403,911 | Ref. | 0.71 (0.32-1.54) | 1.22 (0.60-2.49) | 1.31 (0.64-2.66) |  |

aHR, adjusted hazard ratio; CAD, coronary artery disease; CI, confidence interval; CVD, cardiovascular disease; HbA_1c_, glycated hemoglobin; Hp, haptoglobin

*Models were adjusted for the time-independent variables age, sex, study site, race, education, income, study group assignment, history of CVD at baseline, baseline smoking status, baseline alcohol consumption, diabetes duration, and the time-dependent variables low-density lipoprotein cholesterol, body mass index, diastolic blood pressure, any diabetes medications, any anti-hypertensive medications, and any lipid medication, except for when stratified by one of these variables.

_†_CAD is defined as a composite of the following pre-specified Look AHEAD outcomes: fatal and non-fatal myocardial infarction, hospitalization for angina, and possible fatal CAD.

_‡_P-value for interaction between Hp phenotype and HbA_1c_ categories or between subgroup and HbA_1c_ categories.

**Supplementary Figure 1.** Median high-density lipoprotein (HDL) over study duration did not differ between haptoglobin phenotype groups in either male (top figure) or female (bottom figure) Action for Health in Diabetes (Look AHEAD) participants. Unadjusted median HDL levels in participants measured at baseline and years 1-4, 6, 8, and 10 for Look AHEAD trial participants with the non-Hp2-2 phenotype (blue) and with the Hp2-2 phenotype (orange). Median HDL at each timepoint was compared between Hp phenotype groups using Wilcoxon rank-sum tests.
